# Quality of routine essential care during childbirth: clinical observations of uncomplicated births in Uttar Pradesh, India

**DOI:** 10.2471/BLT.16.179291

**Published:** 2017-04-24

**Authors:** Gaurav Sharma, Timothy Powell-Jackson, Kaveri Haldar, John Bradley, Véronique Filippi

**Affiliations:** a London School of Hygiene & Tropical Medicine, Keppel Street, London, WC1E 7HT, England.; b Sambodhi Research and Communications, New Delhi, India.

## Abstract

**Objective:**

To evaluate the quality of essential care during normal labour and childbirth in maternity facilities in Uttar Pradesh, India.

**Methods:**

Between 26 May and 8 July 2015, we used clinical observations to assess care provision for 275 mother–neonate pairs at 26 hospitals. Data on 42 items of care were collected, summarized into 17 clinical practices and three aggregate scores and then weighted to obtain population-based estimates. We examined unadjusted differences in quality between the public and private facilities. Multilevel linear mixed-effects models were used to adjust for birth attendant, facility and maternal characteristics.

**Findings:**

The quality of care we observed was generally poor in both private and public facilities; the mean percentage of essential clinical care practices completed for each woman was 35.7%. Weighted estimates indicate that unqualified personnel provided care for 73.0% and 27.0% of the mother–neonate pairs in public and private facilities, respectively. Obstetric, neonatal and overall care at birth appeared better in the private facilities than in the public ones. In the adjusted analysis, the score for overall quality of care in private facilities was found to be six percentage points higher than the corresponding score for public facilities.

**Conclusion:**

In 2015, the personnel providing labour and childbirth care in maternity facilities were often unqualified and adherence to care protocols was generally poor. Initiatives to measure and improve the quality of care during labour and childbirth need to be developed in the private and public facilities in Uttar Pradesh.

## Résumé

**Objectif:**

Évaluer la qualité des soins essentiels dispensés lors des accouchements et des naissances sans complications, dans des centres de maternité de l'Uttar Pradesh, en Inde.

**Méthodes:**

Entre le 26 mai et le 8 juillet 2015, nous avons réalisé des observations cliniques pour évaluer les soins dispensés à 275 paires mère/nouveau-né, dans 26 hôpitaux. Des données portant sur 42 soins ont été compilées et synthétisées dans 17 catégories de pratiques cliniques et trois scores globaux, puis pondérées pour obtenir des estimations en population. Nous avons examiné les différences de qualité, avant ajustement, entre les établissements publics et privés. Des modèles linéaires multiniveaux à effets mixtes ont été utilisés afin d'ajuster les données pour tenir compte des caractéristiques des accoucheurs, des centres de maternité et des mères.

**Résultats:**

La qualité des soins que nous avons observés a généralement été médiocre, autant dans le public que dans le privé; le pourcentage moyen de réalisation des soins cliniques essentiels pour chaque femme étant de 35,7%. Les estimations pondérées indiquent que les soins ont été dispensés par du personnel non qualifié dans 73,0% des cas dans les centres publics et dans 27,0% des cas dans les centres privés. Les soins obstétricaux, néonataux et les soins d'ordre général dispensés à la naissance ont semblé de meilleure qualité dans les centres privés que dans les centres publics. Dans notre analyse après ajustement, le score pour la qualité générale des soins dispensés a été supérieur de six points de pourcentage dans les centres privés par rapport aux centres publics.

**Conclusion:**

En 2015, dans les centres de maternité, les soins dispensés lors des accouchements et des naissances ont été réalisés par un personnel souvent non qualifié, et le respect des protocoles de soins s'est généralement avéré médiocre. Des initiatives pour mesurer et améliorer la qualité des soins dispensés lors des accouchements et des naissances doivent être développées dans les centres publics et privés de l'Uttar Pradesh.****

## Resumen

**Objetivo:**

Evaluar la calidad de la atención esencial durante partos y nacimientos normales en maternidades de Uttar Pradesh, India.

**Métodos:**

Entre el 26 de mayo y el 8 de julio de 2015, se utilizaron observaciones clínicas para evaluar la prestación de asistencia a 275 pares madre-neonato en 26 hospitales. Se recopiló información sobre 42 elementos de la atención, resumidos en 17 prácticas clínicas y tres puntuaciones genéricas, y se ponderaron para obtener estimaciones basadas en la población. Se examinaron las diferencias no ajustadas de la atención entre los centros públicos y los privados. Se utilizaron modelos lineales de efectos mixtos en varios niveles para ajustar las características de los asistentes de parto, los centros y las madres.

**Resultados:**

En general, la calidad de la atención observada fue pobre, tanto en los centros públicos como en los privados. El porcentaje medio de las prácticas de atención clínica esencial completadas para cada mujer fue del 35,7%. Las estimaciones ponderadas indican que la atención fue proporcionada por personal no cualificado para el 73,0% y el 27,0% de los pares madre-neonato en centros públicos y privados respectivamente. La atención obstétrica, la atención neonatal y la atención general tras el nacimiento parecieron ser mejor en centros privados que en públicos. En el análisis ajustado, la puntuación para la calidad general de la atención en los centros privados resultó estar seis puntos porcentuales por encima de la puntuación correspondiente a los centros públicos.

**Conclusión:**

En 2015, el personal que proporcionaba atención de parto y nacimiento en maternidades solía carecer de cualificación, y su adherencia a los protocolos de atención era, en general, escasa. Las iniciativas para medir y mejorar la calidad de la atención durante el parto y el nacimiento necesitan desarrollarse en los centros públicos y privados de Uttar Pradesh.

## ملخص

**الغرض:**

تقييم جودة الرعاية الضرورية المقدمة أثناء المخاض العادي والولادة في مراكز الأمومة في ولاية أوتار براديش بالهند.

**الطريقة:**

في الفترة ما بين 26 مايو/أيار، و8 يوليو/تموز 2015، استخدمنا الملاحظات السريرية لتقييم توفير الرعاية لعدد 275 أمًا وأطفالهن في 26 مستشفى. تم جمع البيانات الخاصة بـ 42 عنصرًا من عناصر الرعاية، وتلخيصها في 17 ممارسة سريرية وثلاث نتائج مجمعة ثم ترجيحها للحصول على تقديرات تعتمد على الشرائح السكانية. وقمنا بفحص الاختلافات غير المعدلة في مستوى الجودة ما بين المرافق العامة والخاصة. كما تم استخدام نماذج خطية متعددة المستويات ذات تأثيرات مختلطة لتعديل خصائص المُولّدة والمرافق الصحية والأمهات.

**النتائج:**

كانت جودة الرعاية التي لاحظناها سيئة بوجه عام في المرافق العامة والخاصة على حدٍ سواء؛ حيث بلغت النسبة المئوية لممارسات الرعاية السريرية الضرورية المقدمة لكل امرأة 35.7‏%. وتشير التقديرات الموزونة إلى أن نسبة العاملين غير المؤهلين من مقدمي الرعاية بلغت 73.0‏% و27.0‏% للأم وطفلها في المرافق العامة والخاصة على التوالي. وظهرت رعاية أمراض النساء والولادة ، ورعاية المواليد *نسبة * وإجمالي الرعاية عند الولادة بشكلٍ أفضل في المرافق الخاصة عن مثيلاتها العامة. في التحليل المعدّل، وُجد أن النتيجة الإجمالية للرعاية المقدمة في المرافق الصحية أعلى بست درجات مئوية من الدرجة المماثلة الخاصة بالمرافق العامة.

**الاستنتاج:**

في 2015، كان أغلب العاملين من مقدمي رعاية المخاض والولادة في مرافق الأمومة غير مؤهلين وكان الالتزام ببروتوكولات الرعاية ضعيفًا بوجه عام. ويلزم طرح مبادرات لقياس الجودة المقدمة أثناء المخاض والولادة في المرافق الخاصة والعامة بولاية أوتار براديش. ****

## 摘要

**目的:**

旨在评估印度北方邦地区妇产机构的顺产及正常分娩期基本护理质量。

**方法:**

2015 年 5 月 26 日至 7 月 8 日，我们采用临床观察法对为 26 家医院的 275 对母婴提供的护理进行了评估。期间采集了 42 项护理数据、总结出 17 项临床实践和三项综合得分，然后加权计算出基于人口的估计值。我们审查了公立和私立机构之间护理质量的未调整差异。然后采用多级线性混合效应模型来根据接生员、机构以及产妇特征进行调整。

**结果:**

我们观察到的私立和公立机构的护理质量均普遍较差；每位产妇完成基础临床护理的平均比例为 35.7%。加权估计值表明，公立和私立机构由不具备资格的人员提供护理的母婴对比例分别为 73.0% 和 27.0%；私立机构提供的产妇、新生儿 以及整体分娩护理 优于公立机构。在调整后分析中，私立机构整体护理质量得分比公立机构对应得分高出六个百分点 。

**结论:**

2015 年，妇产机构提供生产和分娩护理的人员经常不具备资格，并且在遵守护理规程方面表现整体较差。北方邦地区的私立和公立机构需要制定相应措施以衡量和提升生产及分娩期护理质量。

## Резюме

**Цель:**

Оценить качество оказания основной медицинской помощи при нормальных родах в родовспомогательных учреждениях штата Уттар-Прадеш, Индия.

**Методы:**

Для оценки оказания медицинской помощи авторы использовали клинические наблюдения 275 пар «мать-новорожденный», проведенные в период с 26 мая по 8 июля 2015 года в 26 больницах. Были собраны данные о качестве оказания медицинской помощи по 42 пунктам, которые были обобщены и представлены в виде 17 клинических практик и трех суммарных оценок, а затем взвешены для получения оценок на основе популяции. Авторы исследовали нескорректированные различия в качестве между государственными и частными медицинскими учреждениями. Для учета характеристик акушеров, медицинских учреждений и матерей были использованы многоуровневые линейные модели смешанных эффектов.

**Результаты:**

Наблюдаемое качество оказания медицинской помощи в целом было низким как в частных, так и в государственных медицинских учреждениях; средняя доля основных клинических процедур, выполненных для каждой женщины, составила 35,7%. Взвешенные оценки показывают, что медицинскую помощь оказывал неквалифицированный персонал для 73,0 и 27,0% пар «мать-новорожденный» в государственных и частных учреждениях соответственно. Было обнаружено, что качество акушерской, неонатальной и общей медицинской помощи при родовспоможении было выше в частных медицинских учреждениях, чем в государственных. В скорректированном анализе оценка общего качества оказания медицинской помощи в частных учреждениях была на шесть процентных пунктов выше, чем соответствующая оценка для государственных учреждений.

**Вывод:**

В 2015 году персонал, оказывающий медицинские услуги при родах в родовспомогательных учреждениях, часто был неквалифицированным и соблюдение протоколов по медицинскому уходу было, как правило, неудовлетворительным. В частных и государственных учреждениях в штате Уттар-Прадеш требуется разработка инициатив по измерению и повышению качества оказания медицинской помощи во время родов.

## Introduction

The quality of care offered at maternity facilities not only affects pregnant women – both emotionally and physically – but also has an impact on the long-term health and survival of mothers and neonates.^1^^,^^2^ An increased focus on care during childbirth can lead to reductions in disability, maternal and neonatal mortality and stillbirths.^2^^,^^3^

An estimated 72% of all deliveries – including 69% of those in South Asia – now occur in health facilities.^4^ Even in health facilities, however, failures in the processes of care can result in bad obstetric and neonatal outcomes^5^^,^^6^ and care of poor quality often leads to low demand for maternal health services.^7^^,^^8^ Some routine interventions can be ineffective or even harmful.^9^

Despite substantial efforts to promote evidence-based obstetrics, the uptake of recommended interventions into clinical practice has been limited.^10^^–^^12^ Clinical practices can be difficult to change because they are influenced by health worker and patient characteristics, the complexity of the tasks involved and the institutional and sociocultural environments.^13^^,^^14^

In 2015, the estimated number of maternal deaths in India was higher than that in any other country apart from Nigeria.^15^ India has to make rapid improvements in its levels of maternal mortality if the Global Strategy for Women’s, Children’s and Adolescents’ Health’s targets are to be met by 2030.^16^ Maternity services in India are available from an enormous range of health providers. Maternity care in the public sector is provided through a network of primary, secondary and tertiary facilities that, in principle, provide routine care, basic emergency obstetric care and comprehensive emergency obstetric care, respectively.^17^ In the private sector, maternity care is provided by a heterogeneous collection of facilities that range from small maternity homes to large multispecialty medical colleges and tertiary hospitals.

An analysis of the results of Demographic and Health Surveys conducted in 57 countries between 2000 and 2013 revealed that, in the various regions of the world, the private sector accounted for 9−56% of deliveries.^18^ In 2003–2005, an estimated 22% of all deliveries in India occurred in the private sector.^19^ Among Indian women, previous negative pregnancy outcomes and relatively high socioeconomic status are positively associated with use of private facilities^19^ whereas belonging to a so-called scheduled caste or tribe is negatively associated with such use.^20^ The private sector is more expensive than the public sector but most Indians associate the private sector with better amenities and a higher standard of care.^20^

Although much information exists on the quality of emergency obstetric care in India,^21^^,^^22^ there appears to have been little research on the quality of normal labour and childbirth care, particularly in private facilities. The results of a few relevant qualitative studies on the public sector have generally revealed care of poor quality, often characterized by high rates of labour augmentation, routine episiotomies, no choice of position, non-adherence to protocols, limited monitoring, early discharge from the hospital and poor neonatal care.^23^^–^^25^ In most areas of the world, deliveries in the private sector are much more likely to be by caesarean section than deliveries in the public sector.^26^^–^^29^ This paper reports findings from clinical observations that were used to describe and investigate the quality of care provided routinely, for uncomplicated labour and childbirth, in maternity facilities in Uttar Pradesh, India.

## Methods

### Study setting

This study was conducted in three districts of Uttar Pradesh: Kannauj, Kanpur Dehat and Kanpur Nagar.^30^ In 2012–2013, Uttar Pradesh was the Indian state with the largest population and the second and third highest levels of maternal and neonatal mortality, respectively.^31^ At this time, the estimated number of neonatal deaths per 1000 live births was 55 deaths in Kannauj, 41 deaths in Kanpur Dehat and 24 deaths in Kanpur Nagar. The estimated percentage of deliveries occurring in public and private facilities, respectively, was 43% and 15% in Kannauj, 46% and 10% in Kanpur Dehat, and 34% and 34% in Kanpur Nagar.^31^ Also widespread inequities across the continuum of care existed – in terms of the recorded indicators of maternal, neonatal and reproductive health – in the three study districts.^31^

### Sampling

We used a multistage sampling method. The initial sampling frame included 59 facilities in Uttar Pradesh that provided maternity services: all 29 of the larger public facilities listed by the Indian Department of Health – i.e. facilities that reported at least 200 deliveries per month^32^ and, in theory, provided basic emergency obstetric care at all hours of the day and night – plus the 30 private facilities that, in theory, provided continuous maternity care. The private facilities were identified by key informants from Sambodhi Research and Communications (Lucknow, India) – an organization that has worked in health research in the study districts for several years.

**Fig. 1:**
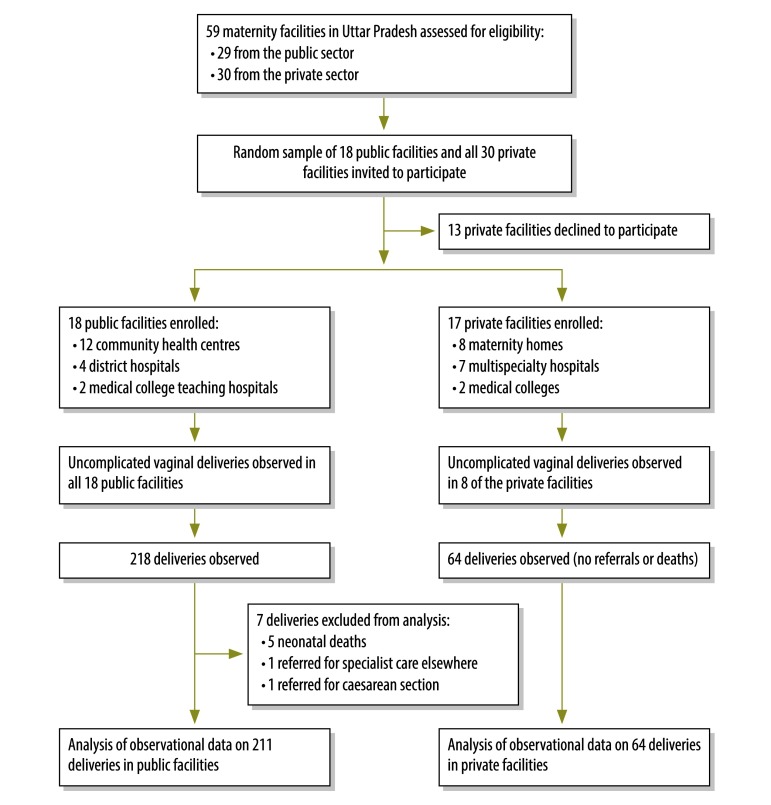
Flowchart showing the selection and investigation of participants for the study of the quality of maternal and neonatal care at birth, Uttar Pradesh, India, 2015

In the second stage of sampling, we attempted to select six public facilities per district – i.e. a random selection of four of the community health centres, one of the medical colleges and one of the district hospitals. Since Kanpur Dehat did not have a medical college, we had to select an additional district hospital. Although we invited the 18 selected public facilities and all 30 private facilities to participate in our study, 13 facilities – all private – refused to participate. At nine of the facilities that agreed to participate – again all from the private sector – no deliveries occurred while observers were present. The observational data that we analysed therefore came from 18 public facilities and eight private (Fig. 1). Power calculations were used to estimate the number of observations required at each facility (available from the corresponding author). We expected observations of up to 10 deliveries to be completed either over the two days of observation at each public facility or over the week of observation at each private facility. The 211 observed deliveries from 18 public sector facilities are a sample of an estimated 41, 512 annual deliveries that occurred in 18 public sector facilities in 2015.  The 64 observed deliveries from eight private sector facilities represented 3 579 deliveries from 8 private sector facilities in 2015. These data on annual caseloads were self-reported by health facilities and collected by us during the study. The larger household survey in three study districts found that public sector deliveries account for 54.8% (*n *=1 943), private sector account for 13.7% (*n *= 486) and home deliveries account for 31.5% (*n *=1 117) annually. The public sector was found to be 3.98 times larger than the private sector. Therefore, to get a representative sample of births by health facility, we multiplied the private sector births by a factor of 2.94 to get a total of 10 535.

### Study participants and sites

Study participants were pregnant women with spontaneous, uncomplicated labours who gave their written informed consent. Pregnant women were enrolled if they had a gestational age between 37 and 42 weeks and a singleton pregnancy with vertex presentation. We observed the post-admission care provided to these women and their neonates until one hour postpartum.

### Data collection

We developed an assessment tool (available from the corresponding author) based on a critical assessment of previously tested instruments^12^^,^^33^ and the relevant World Health Organization guidelines.^34^ Questions capturing demographic, educational and socioeconomic status were adapted from the National Family Health Survey questionnaire.^35^ At maternity facilities, 14 trained enumerators with a clinical background visited the admissions, emergency, labour and postnatal wards to identify pregnant women who were likely to undergo uncomplicated vaginal births. Two enumerators were then stationed at each facility for either two days – if the facility was in the public sector – or a week – if the facility was in the private sector – and they observed round-the-clock provision of care for mothers and their neonates. Data were collected between 26 May and 8 July 2015.

### Ethics

We obtained ethical approval from the Ethics Review Board of the Public Health-care Society and the Indian Council for Medical Research in India, and the London School of Hygiene & Tropical Medicine in the United Kingdom of Great Britain and Northern Ireland.

### Measures

Learning from previous quality measurement efforts,^36^^,^^37^ our assessments of quality of care encompassed not only the provision of clinical care but also clients’ experiences of care. We investigated both the application of evidence-based practices – including use of potentially harmful interventions – and woman-centred respectful care practices during the birthing process.^38^ We collected data on 42 items of care for each observation (Table 1). Each item was coded 1 if completed and 0 if not. We then aggregated the items into 17 care practices – i.e. nine obstetric and eight neonatal – and scored each practice 1 if fully completed and 0 if not (Table 1). Some practices were based on a single item and some were based on multiple items. Finally, summary scores for obstetric care, neonatal care and overall essential care at birth – based on the relevant nine, relevant eight and all 17 clinical practices, respectively – were calculated as the percentage of the practices measured that were completed for each woman.

**Table 1:** Framework used for the assessment of essential care at birth, India, 2015

Timing	Obstetric care			Fetal or neonatal care	
	Clinical practice	Observed items		Clinical practice	Observed items
On admission and during first stage of labour	Regular monitoring of labour using a partograph	Is labour monitored regularly with partograph?		Check fundal height and fetal presentation	Is fundal height checked and is fetal presentation checked?
	Measures for the prevention of maternal infection during admission	Are hands washed before examination and are sterile gloves put on before vaginal examination?		Regular monitoring of fetal heart rate	Is fetal heart rate monitored at regular intervals?
	Screening for pre-eclampsia and eclampsia	Is blood pressure monitored and urine tested for proteins?			
From second stage of labour to completion of childbirth	Measures for the prevention of maternal infection during childbirth	Are sterile gloves put on before vaginal examination and are vulva and perineum cleaned with antiseptic?		Health workers prepared for resuscitation if required	Is ventilation bag available and is neonatal mask available and laid out?
	Active management of the third stage of labour	Is uterotonic given within minute of birth, is the cord clamped and is there controlled cord traction?		Neonatal cord care	Is cord cut with a sterile instrument?
	Assessment of maternal blood loss	Are the placenta and membranes checked for completeness, is the vagina checked for tears and is there monitoring of bleeding postpartum?		Appropriate thermal care of neonate	Is neonate dried properly; is skin-to-skin contact between neonate and mother initiated and is the neonate covered with a dry towel?
	Use of woman-centred respectful care practices	Is process of labour explained to the mother or support person at least once, is companion allowed to be with the mother during labour, is mother informed before vaginal examination, is visual privacy ensured and is mother asked about choice of position?		Assessment of Apgar score	Is the Apgar score assessed one minute after birth and is it assessed five minutes after birth?
				Initiation of early breastfeeding	Did the mother initiate breastfeeding within hour of birth?
	Avoidance of harmful or unnecessary interventions for mother	Is an enema given, is the pubic area shaved, is fundal pressure applied to hasten delivery of baby or placenta, is there uterine lavage after delivery, is there manual exploration of the uterus after delivery and is there use of episiotomy without any indication?		Avoidance of harmful or unnecessary practices for neonate	Is their routine aspiration of neonate’s nose, is the neonate slapped and is the neonate held upside down?
	Avoidance of harmful or unnecessary health worker behaviour	Does the health worker restrict mother’s fluid and food intake during labour; do they insult, shout or threaten the mother during labour and childbirth; and, do they hit, pinch or slap the mother during labour and childbirth?			

Note: We assessed nine obstetric care and eight neonatal care practices.

For each woman investigated, data on household ownership of a common set of assets were collected and then used, in principal components analysis, to generate quintiles of wealth status.^39^ We recorded the age, caste, day and time of admission, parity, referral status and wealth quintile of each woman, whether the birth attendants were qualified or unqualified and the facilities’ maternity caseloads – i.e. the numbers of deliveries recorded in 2014.

### Analysis

Descriptive analyses were carried out at the level of individual women. We used the *svy* command in Stata version 14 (StataCorp. LP, College Station, United States of America) to account for clustering and to incorporate weights based on each facility’s maternity caseload. All of the percentages shown in the Results section are weighted estimates. Frequencies, means, prevalence and proportions were calculated for covariates disaggregated by sector. A two-level linear mixed-effects model was used – with a random effect at the facility level to account for clustering.^40^ The exposure variable was public or private sector. The explanatory variables were the birth attendant’s and women’s characteristics and the maternity caseloads that we had recorded and – to reduce the effects of any inter-observer bias – a dummy variable for each enumerator. Estimation was by restricted maximum likelihood. We used a Wald test to generate an overall *P*-value for each categorical variable – e.g. age group – and assess whether there was a significant association between a given explanatory variable and the quality of care that had been observed.

## Results

### Sample characteristics

Of the 275 observations, 211 were conducted in public facilities and most pregnant women had come directly to the study facilities (91.5%), were 20 to 34 years of age (90.4%), multiparous (56.0%) and belonged to the caste category known as ‘other backward’ (51.4%; Table 2). Compared with those in the public sector, higher proportions of pregnant women in the private sector belonged to the caste category known as ‘other backward’ (*P* = 0.002) and – although not statistically significant – to the wealthiest quintile (*P* = 0.07) (Table 2). According to the weighted estimates, qualified personnel performed 73.0% of deliveries in the private sector but only 27.0% of those in the public sector (*P* = 0.01) and 99.5% of maternity cases seen in the private sector but only 93.1% of those seen in the public sector were admitted during daytime work-hours (*P* = 0.003; Table 2).

**Table 2:** Characteristics of pregnant women with uncomplicated births investigated in public and private maternity facilities, Uttar Pradesh, India, 2015

Characteristic	Unweighted numbers (%)				Weighted percentages^a^			*P*^b^
	Total (*n* = 275)	Public (*n* = 211)	Private (*n* = 64)		Total (*n* = 52 047)	Public (*n* = 41 512)	Private (*n* = 10 535)	
**Age in years**								0.85
< 20	16 (5.8)	12 (5.6)	4 (6.2)		5.5	5.7	4.4	
20 to 34	247 (89.8)	191 (90.5)	56 (87.5)		90.4	90.4	90.5	
≥ 35	12 (4.3)	8 (3.7)	4 (6.2)		4.1	3.8	5.1	
**Parity**								0.3
Primipara	119 (43.2)	90 (42.6)	29 (45.3)		44.0	41.6	53.4	
Multipara	156 (56.7)	121 (57.3)	35 (54.7)		56.0	58.4	46.6	
**Referral status**								0.003
Came directly to study facility	243 (88.4)	197 (93.4)	46 (71.9)		91.5	95.9	74.1	
Referred from another facility	32 (11.6)	14 (6.6)	18 (28.1)		8.5	4.0	25.9	
**Caste category**								0.002
Scheduled caste	59 (21.4)	53 (25.1)	6 (9.4)		24.2	28.7	6.4	
Scheduled tribe	2 (0.7)	0 (0.0)	2 (3.1)		0.3	0.0	1.4	
Other backward caste	153 (55.6)	111 (52.6)	42 (65.6)		51.4	48.9	61.1	
General caste	61 (22.2)	47 (22.3)	14 (21.8)		24.1	22.3	31.0	
**Wealth quintile**								0.07
First (poorest)	56 (20.4)	49 (23.2)	7 (11.0)		22.5	24.2	15.9	
Second	54 (19.6)	46 (21.8)	8 (12.5)		17.7	19.5	10.6	
Third	55 (20.0)	36 (17.0)	19 (29.6)		17.7	17.6	18.2	
Fourth	55 (20.0)	46 (21.8)	9 (14.0)		19.5	21.9	9.9	
Fifth (wealthiest)	55 (20.0)	34 (16.1)	21 (32.8)		22.5	16.7	45.3	
Type of birth attendant								0.01
Qualified^c^	113 (41.1)	75 (35.5)	38 (59.4)		36.2	27.0	73.0	
Unqualified^d^	162 (58.9)	136 (64.5)	26 (40.6)		63.8	73.0	27.0	
**Timing of admission**								0.003
Within daytime work-hours^e^	254 (92.3)	191 (90.5)	63 (98.4)		94.4	93.1	99.5	
Out of hours	21 (7.6)	20 (9.5)	1 (1.5)		5.5	6.9	0.5	
**Admission day**								0.58
Weekday	211 (76.7)	158 (74.8)	53 (82.8)		77.2	75.9	81.9	
Saturday or Sunday	64 (23.3)	53 (25.1)	11 (17.1)		22.8	24.0	18.1	

^a^ Weighted according to the reported maternity caseload of the study facilities in 2014.

^b^ For the comparison of the weighted percentages for the private sector with the corresponding values for the private sector.

^c^ Doctors, nurses or nurse-midwives – with at least 5, 4 and 2 years of pre-service training, respectively – who are licensed, regulated and endorsed by the government to provide maternity care at health facilities.

^d^ Accredited social health activists, cleaners, hospital porters, other community health workers, traditional birth attendants and others who are not legally allowed by the government to provide maternity care at health facilities.

^e^ That is, between 09:00 and 17:00.

### Care quality by sector

Table 3 shows the quality of care by sector – in terms of each of the clinical practices measured. In the overall provision of obstetric care, in both sectors, monitoring of labour using a partograph (1.7%), screening for pre-eclampsia or eclampsia (2.3%), woman-centred care (3.5%), avoidance of harmful and/or unnecessary interventions (4.3%) and the active management of the third stage of labour (24.5%) were relatively rare whereas measures for the prevention of maternal infection during admission (76.4%) and health worker avoidance of behaviours harmful to the mothers (74.2%) were common. In the provision of obstetric care, assessment of maternal blood loss (*P* = 0.01), measures for the prevention of maternal infection during childbirth (*P* = 0.05) and partograph use (*P* < 0.001) were observed significantly more frequently in the private sector than in the public sector.

**Table 3:** Clinical practices and overall measures of quality in public and private maternity facilities in Uttar Pradesh, India, 2015

Practice	Unweighted numbers (%)							Weighted percentages**^a^**					
	Total (*n* = 275)	Public (*n* = 211)	Public sector 95% CI**^b^**	Private (*n* = 64)	Private sector 95% CI	*P***^c^**		Total (*n* = 52 047)	Public (*n* = 41 512)	Public sector 95% CI	Private (*n* = 10 535)	Private sector 95% CI	*P***^c^**
**For obstetric care**													
Regular monitoring of labour using a partograph	3 (1.1)	1 (0.5)	0.1 to 3.3	2 (3.1)	0.8 to 11.8	0.07		1.7	0.3	0.0 to 2.0	7.2	1.7 to 25.9	< 0.001
Measures for the prevention of maternal infection during admission	212 (77.0)	159 (75.4)	69.0 to 80.7	53 (82.8)	71.4 to 90.3	0.21		76.4	73.4	65.5 to 80.0	88.2	76.8 to 94.4	0.1
Screening for pre-eclampsia and eclampsia	3 (1.1)	2 (0.9)	0.2 to 3.7	1 (1.5)	0.2 to 10.5	0.67		2.3	2.2	0.5 to 9.3	2.5	0.3 to 15.9	0.9
Measures for the prevention of maternal infection during childbirth	115 (41.8)	76 (36.0)	29.8 to 42.8	39 (60.9)	48.4 to 72.2	< 0.001		45.6	38.3	31.0 to 46.2	74.1	59.3 to 84.9	0.05
Active management of the third stage of labour	73 (26.5)	58 (27.4)	21.9 to 33.9	15 (23.4)	14.6 to 35.5	0.52		24.5	25.4	19.3 to 32.5	21.2	11.4 to 36.1	0.7
Assessment of maternal blood loss	124 (45.1)	81 (38.4)	32.0 to 45.2	43 (67.2)	54.7 to 77.6	< 0.001		42.8	34.5	27.4 to 42.4	75.7	60.7 to 86.2	0.01
Use of woman-centred respectful care practices	12 (4.4)	9 (4.3)	2.2 to 8.0	3 (4.7)	1.5 to 13.7	0.88		3.5	2.9	1.4 to 5.8	5.6	1.1 to 24.7	0.5
Avoidance of harmful or unnecessary interventions for mother	15 (5.4)	14 (6.6)	4.0 to 10.9	1 (1.5)	0.2 to 10.5	0.12		4.3	5.0	2.9 to 8.6	1.5	0.2 to 10.2	0.2
Avoidance of harmful or unnecessary health worker behaviour	215 (78.2)	162 (76.7)	70.6 to 82.0	53 (82.8)	71.4 to 90.3	0.30		74.2	72.4	64.2 to 79.3	81.2	57.3 to 93.3	0.45
**For fetal or neonatal care**													
Check of fundal height and fetal presentation	4 (1.4)	1 (0.5)	0.1 to 3.3	3 (4.7)	1.5 to 13.7	0.014		1.1	0.5	0.1 to 3.7	3.4	0.8 to 14.1	0.08
Regular checking of fetal heart rate	61 (22.2)	20 (9.5)	6.2 to 14.3	41 (64.0)	51.5 to 74.9	< 0.001		20.1	6.6	4.1 to 10.5	73.3	58.5 to 84.2	< 0.001
Health workers prepared for resuscitation if required	179 (65.1)	132 (62.6)	55.8 to 68.9	47 (73.4)	61.2 to 82.9	0.11		68.1	67.2	60.0 to 73.7	71.6	51.2 to 85.8	0.8
Neonatal cord care	265 (96.4)	202 (95.7)	92.0 to 97.8	63 (98.4)	89.5 to 99.8	0.3		95.2	94.6	88.7 to 97.6	97.5	84.0 to 99.7	0.5
Appropriate thermal care of neonate	84 (30.5)	62 (29.4)	23.6 to 35.9	22 (34.4)	23.7 to 46.9	0.4		37.7	36.5	29.0 to 44.8	42.4	24.8 to 62.1	0.7
Assessment of Apgar score	1 (0.36)	0 (0.0)	0.0 to 0.0	1 (1.5)	0.2 to 10.5	0.07		0.9	0.0	0.0 to 0.0	4.7	0.7 to 26.8	0.08
Initiation of early breastfeeding	191 (69.4)	148 (70.1)	63.6 to 76.0	43 (67.2)	54.7 to 77.6	0.6		69.8	70.9	62.4 to 78.1	65.6	48.7 to 79.3	0.6
Avoidance of harmful or unnecessary practices for neonate	95 (34.5)	70 (33.2)	27.1 to 39.8	25 (39.0)	27.8 to 51.6	0.3		38.0	35.3	27.9 to 43.6	48.8	31.3 to 66.6	0.3
**Aggregate indices of quality of care **													
Obstetric care	275 (31.2)	211 (29.6)	27.9 to 31.3	64 (36.5)	33.4 to 39.6	0.03		30.6	28.3	25.9 to 30.5	40.0	35.4 to 44.0	0.01
Neonatal care	275 (40.0)	211 (37.6)	36.1 to 39.2	64 (47.8)	44.1 to 51.6	0.02		41.4	39.0	37.2 to 40.7	51.0	44.8 to 57.0	0.02
Essential care at birth	275 (35.3)	211 (33.4)	32.0 to 34.7	64 (41.8)	38.9 to 44.7	0.01		35.7	33.3	31.6 to 35.0	45.0	40.5 to 49.5	0.01

CI: confidence interval.

^a^ Weighted according to the reported maternity caseload of the study facilities in 2014.

^b^ Percentage values.

^c^ For the comparison of the estimates for the private sector with the corresponding values for the private sector.

In the provision of fetal or neonatal care across both sectors, assessment of Apgar scores one and five minutes after birth (0.9%), assessment of fetal presentation and fundal height (1.1%) and the regular monitoring of fetal heart rate (20.1%) were rare whereas resuscitation preparedness (68.1%), sterile cord care (95.2%) and support for early initiation of breastfeeding (69.8%) were relatively common. One clinical practice – the regular monitoring of fetal heart rate – was observed much more frequently in the private sector than in the public sector (73.3% vs 6.6%; *P* < 0.001). Observational data disaggregated by each of the 42 items of care that were observed are available from the corresponding author.

Quality of essential care during labour and childbirth was found to be deficient (mean: 35.7%) across our entire sample of facilities (Table 3). Overall, 45.0% of recommended clinical practices were completed among women giving birth in the private sector compared with 33.3% in the public sector (*P* = 0.01). Private-sector clients received 40.0% of the recommended obstetric care practices and 51.0% of the recommended neonatal care practices – compared with 28.3% (*P* = 0.01) and 39.0% (*P* = 0.02), respectively, in the public sector.

The results from the multivariate analysis revealed that, after controlling for confounders, the overall quality of care score was six percentage points higher (*P* = 0.03) in the private sector than in the public sector (Table 4). We found no association between use of qualified personnel, facility caseload or the woman’s age, caste, parity, referral status or socioeconomic status and the overall quality of care at the time of birth. However, compared with admission on a weekday, admission during the weekends was associated with a quality of care score that was three percentage points lower (*P* = 0.03).

**Table 4:** Investigation of the association between the index for the quality of essential care at birth and the characteristics of the birth attendants, maternity facilities and mothers, Uttar Pradesh, India, 2015

Explanatory variable	Coefficient**^a^ (**95% CI)	*P*
**Characteristics of birth attendant**		0.61
Unqualified	Base	
Qualified	0.01 (−0.02 to 0.04)	
**Characteristics of facility**		
Facility sector		0.03
Public	Base	
Private	0.06 (0.01 to 0.11)	
No. of deliveries at facility in 2014		0.77
< 2000	Base	
2000 to 2999	0.01 (−0.05 to 0.06)	
≥ 3000	−0.02 (−0.08 to 0.05)	
**Characteristics of mother**		
Day of admission		0.03
Weekday	Base	
Saturday or Sunday	−0.03 (−0.06 to 0.003)	
Age in years		0.91
< 20	Base	
21 to 34	0.01 (−0.04 to 0.05)	
≥ 35	0.01 (−0.05 to 0.08)	
Parity		0.22
Primipara	Base	
Multipara	0.01 (−0.01 to 0.03)	
Referral status		0.84
Came directly to study facility	Base	
Referred from another facility	0.00 (−0.04 to 0.03)	
Caste		0.15
Scheduled caste or scheduled tribe	Base	
Other backward caste	0.02 (−0.01 to 0.04)	
General caste	0.03 (0.00 to 0.06)	
Wealth quintile		0.08
First (poorest)	Base	
Second	0.00 (−0.03 to 0.03)	
Third	0.00 (−0.03 to 0.03)	
Fourth	0.00 (−0.03 to 0.03)	
Fifth	0.04 (0.00 to 0.07)	
Timing of admission		0.62
Within daytime work-hours^b^	Base	
Out of hours	−0.01 (−0.05 to 0.03)	

CI: confidence interval.

^a^ Results from a multilevel mixed-effects linear regression.

^b^ That is, between 09:00 and 17:00.

When we examined adjusted variances, for quality of care, between health workers, we found greater variation within health workers (standard deviation, SD: 0.004) than between them (SD: 0.002; intraclass correlation: 0.33). Similarly, there was greater variation, for quality of care, within health facilities (SD: 0.005) than between them (SD: 0.002; intraclass correlation: 0.27). We found no evidence that birth attendants were exerting more – or less – effort simply because they were being observed and there was, therefore, no significant Hawthorne effect (available from the corresponding author).

## Discussion

Using clinical observations, we found that, in Uttar Pradesh, essential care provided to women and their neonates – during labour and childbirth – was generally of poor quality. The private facilities generally outperformed the public facilities in terms of both obstetric and neonatal care. Measures to prevent some major causes of maternal mortality – e.g. haemorrhage, hypertensive disorders and sepsis – were rare in both the private and public sectors.

Our study advances the descriptive evidence base on quality of care at the time of birth in India – particularly for the private sector, which has an increasing share of the market for maternity care.^18^ Direct observations of clinical practices offer advantages over other methods of quality assessment, especially when – as in our study – there is no evidence of a Hawthorne effect. We developed a comprehensive measure of quality of care that included adherence to evidence-based guidelines, respectful care practices, harmful and unnecessary interventions and harmful health worker behaviours. The methods we used to calculate separate indices for neonatal care, obstetric care and overall essential care at birth could be used for monitoring quality of care in other settings.

Our multivariate analysis confirmed that, in our study districts, private maternity facilities generally provided a higher standard of care than those in the public sector and that the quality of care provided – in either sector – was not significantly related to the investigated characteristics of the birth attendant, facility or the woman’s age, caste, parity, referral status or socioeconomic status. However, compared with admission at other times, admission at a weekend was associated with poorer quality of care. Other studies have also revealed poorer neonatal and obstetric care during weekends than at other times.^41^^,^^42^

Care during labour and childbirth in the public sector was less likely to be provided by qualified staff than such care in the private sector. However, we did not find that care provided by qualified personnel was significantly better than that provided by unqualified personnel. Even qualified birth attendants may not be adequately skilled.^25^^,^^43^ In a study from India using standardized patients, only minor differences were found between the quality of care given by trained providers and that given by untrained providers – although this study did not focus on maternal and neonatal care.^44^

We did not find any relationship between facility size and quality of care at birth – perhaps because our observations were limited to uncomplicated vaginal births and quality of care for such births was generally poor irrespective of the facility caseload. Previous studies have found a relatively better quality of care at large high-level facilities and this may explain why patients may sometimes bypass small low-level facilities.^7^ Although, we do not have robust evidence on the factors influencing quality of care at maternity facilities in India, evidence from low-income countries indicates that provider effort could be a key determinant.^45^ Evidence also exists that the private sector generally provides better quality of care because it has superior management and operational systems – including better incentive schemes that attract more motivated and better qualified staff.^44^

Our findings are similar to those of some other studies in India. In a study based in Rajasthan, partograph use was found to be especially weak and monitoring was found often to consist only of repeated unhygienic vaginal examinations.^24^ We found active management of the third stage of labour to be more common in the facilities we surveyed than reported in some neighbouring districts of Uttar Pradesh.^23^ We found respectful rights-based maternity care^38^ to be rare. Our informal observations during data collection – of labour room environments that often appeared chaotic and of some health workers that could be abusive, dominating and threatening on occasions (available from the corresponding author) – were consistent with those previously found in Madhya Pradhesh^46^ and Rajasthan.^25^ Inadequate knowledge and skills, lack of enabling environments, limited supportive supervision, staffing shortages and the poor quality of in-service training could all be underlying causes of the generally poor quality of maternity care in India.^24^^,^^46^ The Indian government is currently implementing a range of schemes to improve the quality of intrapartum and immediate postpartum care.^47^ Given the shortages of skilled human resources for maternity care in India, focused efforts to establish a professional cadre of midwives could be beneficial. We found greater variance in quality of care within individual health workers than between them. This could indicate that health workers do not follow standard protocols and/or provide preferential care.

Our study had several limitations. First, there may have been observer bias – e.g. due to the general perception that the private sector is superior because it has better infrastructure and better trained personnel. Second, there were challenges in sampling the private sector. Not only did 13 private facilities refuse to participate but also we had no official sampling frame from which to select private facilities. It is possible that the quality of care provided by the participating private facilities was different to that provided by the other private facilities in Uttar Pradesh. Third, although it provided useful summary measures, our aggregation of numerous indicators into broader indices will have masked variation between individual indicators. Also, in developing our aggregate measures of quality, we gave equal weight to each indicator because there was no scientific basis for applying intervention-specific weights. All of the women who were invited to participate in the study agreed to participate and, by following a strict case-definition, we hoped to minimize any selection bias at participant level. To limit subjectivity, our observers were well trained and used a structured questionnaire to record their observations.

Our findings have at least three key implications. First, a systematic effort to measure and identify existing quality gaps during labour and childbirth, is warranted, especially in India’s high-burden states. Such research should include private-sector facilities, which provide a substantial and increasing proportion of the maternity care in India. Second, the reasons for the high prevalence of maternity care provided by untrained personnel and the widespread non-adherence to recommended protocols should be investigated further. Third, tailored quality-improvement initiatives^48^ must be designed for facilities in both sectors – with the regular auditing of the actual processes of care linked to functional accountability mechanisms.
